# Supplementary figures and images for: High-throughput library transgenesis in Caenorhabditis elegans via Transgenic Arrays Resulting in Diversity of Integrated Sequences (TARDIS)
Source: eLife. 2023 Jul 4;12:RP84831. doi: 10.7554/eLife.84831 (PMC10328503; doi:10.7554/eLife.84831)

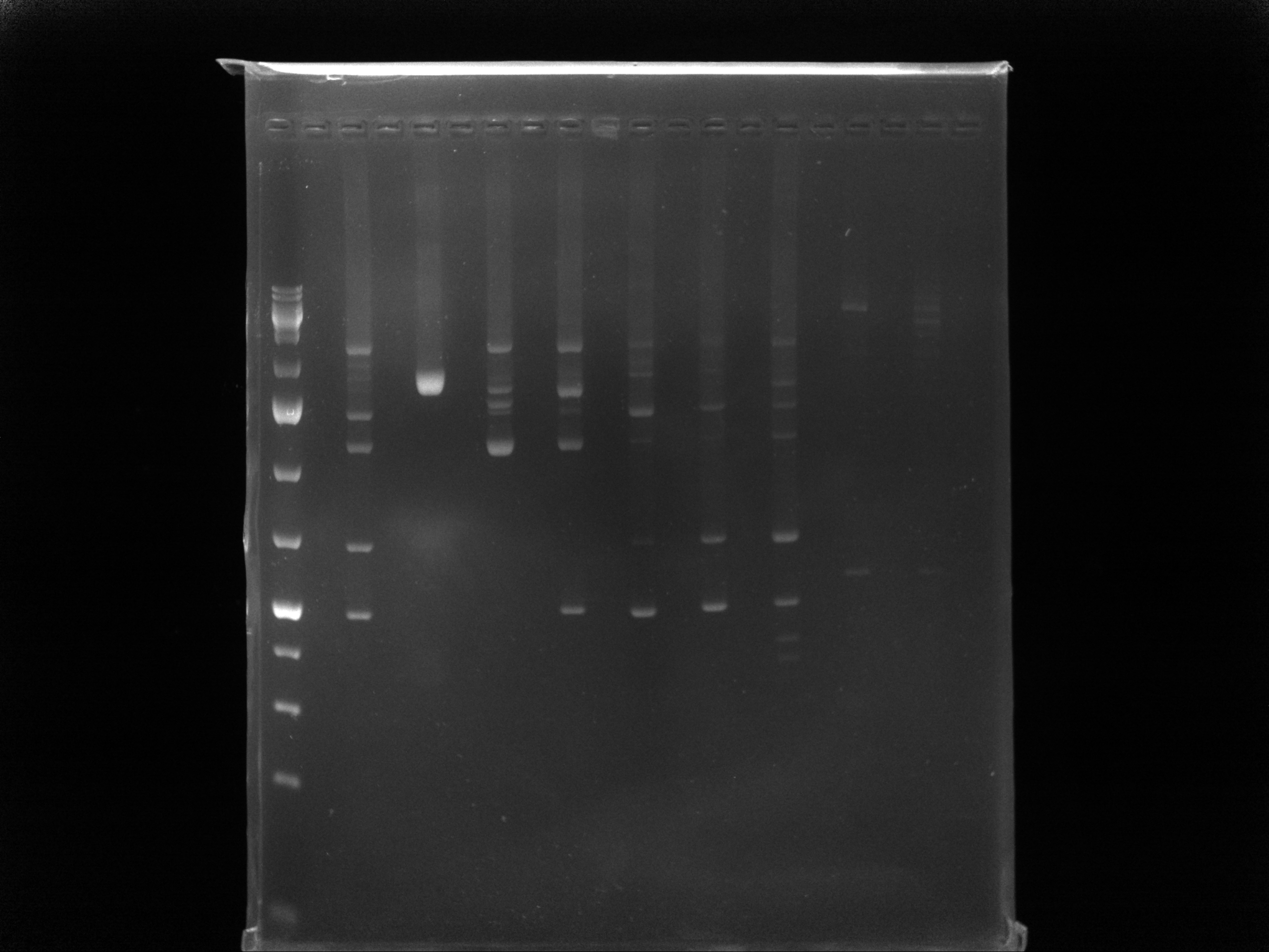

Supplement: Figure 5—figure supplement 1—source data 1. [file elife-84831-fig5-figsupp1-data1.zip › Figure 5-figure suppplement 1-source data 1/Figure5_S1A_unedited.tif]

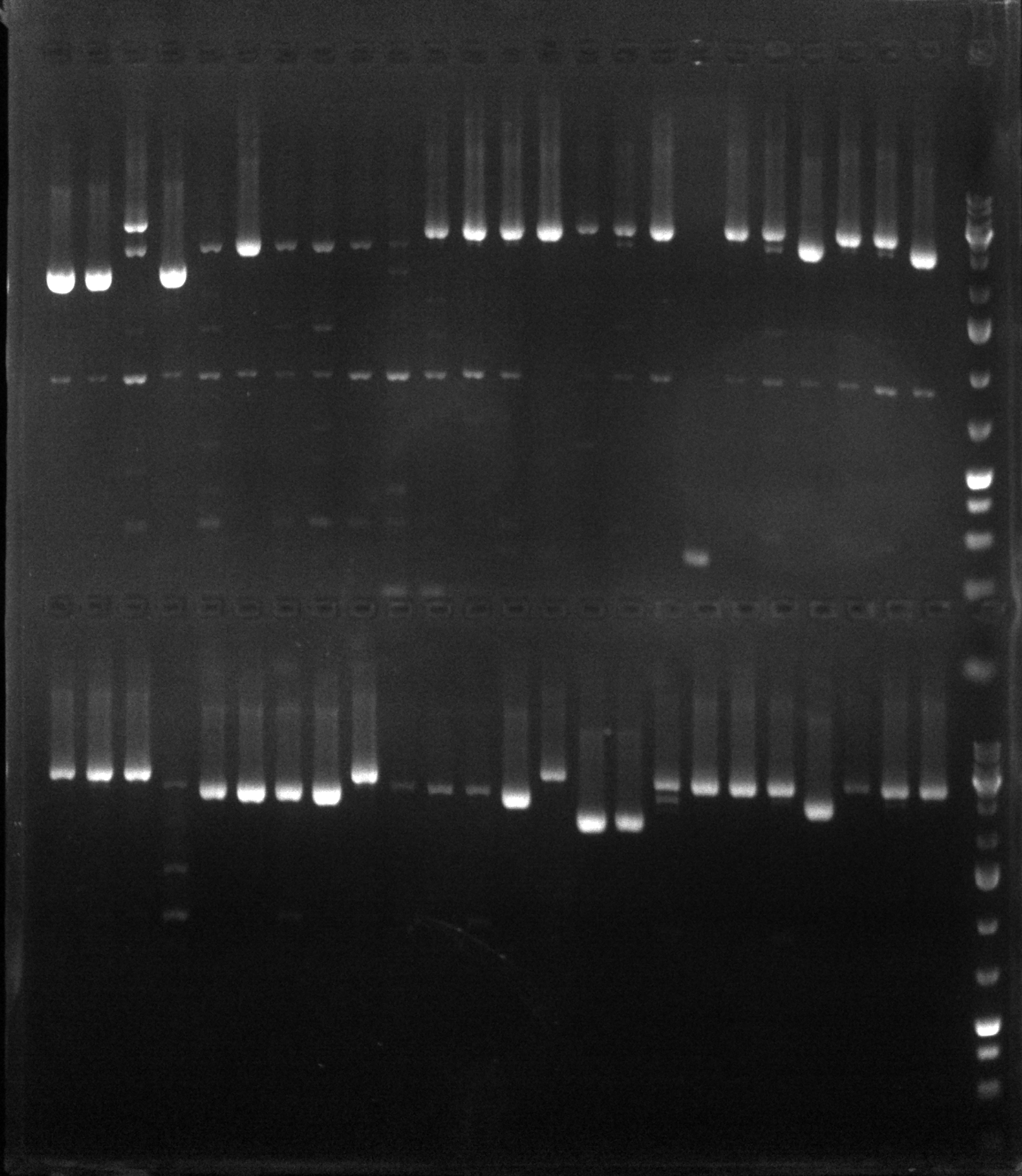

Supplement: Figure 5—figure supplement 1—source data 1. [file elife-84831-fig5-figsupp1-data1.zip › Figure 5-figure suppplement 1-source data 1/Figure5_S1C_unedited.tif]
